# Treatment of Fistula in Ano

**Published:** 1856-02

**Authors:** 


*Treatment of Fistula in Ano.* By T. R. Mitchell, M. D., F. R. C. S. I.

I am anxious to direct the notice of the profession to a modification of treatment in the cure of fistula in ano. It is, I believe, an established axiom that in all cases it is necessary to divide the sphincter ani muscle, the usual operation consisting of the introduction of a probe-pointed pistoury as far as the sinus extends. Others recommend the mere division of the sphincter in a lower situation. Whichever operation is performed, the result is very often a relaxed state of the sphincter for some time afterwards.

In a case on wh ch I operated twelve months ago, the gentleman had considerable difficulty in retaining the bowel up, particularly after violent exercise or defecation. In this case I found that the fibres of the spincter ani were much relaxed, so much so as to allow the rectum to protrude for several inches, so as very closely to resemble a prolapsed uterus. The constitution began to suffer from the constant discharges of muco-purulent matter, and he was quite incapacitated from following his employment. Having prviously emptied the rectum by an aperient, I directed him to force down as much as possible, and then proceeded to touch the tumor with strong nitric acid; this was done with a piece of thin wood, four stripes extending from the upper part of the tumor to the sphincter being made on the surface; the part was then smeared well over with oil, and returned. The operation required to be repeated at the end of ten days, when only about two inches of the rectum could be forced down, and he has since then been able to go about his employment without the slightest inconvenience.

The profession are indebted to the late Dr. Houston, of Dublin, for the introduction of nitric acid in the treatment of vascular tumors of the rectum, many cases of its successful employment being given by him in the twenty-third volume of the *Dublin Journal of Medical Science.*

Since the above case was treated, I have had several of a similar nature, and the result has been the same. It would, therefore, be unpardonable in me to enlarge further on the subject, particularly as a similar treatment has been adopted very extensively by other surgeons, and is well known to the profession. It, however, struck me that if, instead of the great relaxation of the sphincter which so frequently follows its division, we could cause a constriction as great or nearly so as before the operation, we should be doing good service. Now this I think may be accomplished by a very simple method—employing the nitric acid before the relaxation takes place, or prior to any protrusion; and the plan I adopt, and which I have hitherto found very successful, is to apply the strong nitric acid around the margin of the sphincter ani which have been divided, and this I do on the fourth day after the operation; the pain of its application is quickly removed by smearing the parts over with oil, and it is only necessary to apply it twice.

Before concluding these remarks, I wish to state that I have found patients laboring under diseases of the rectum particularly difficult to get under the influence of chloroform, and have found the process much facilitated by employing it locally as well as by inspiration, as I have found the parts excessively sensitive even when the patient has apparently been fully under its influence, and when pricking or pinching was unheeded. This, I think, may be easily explained by the fact of the patient's sufferings having been for some time directed to the part, and to the nerves being in a highly sensitive condition.—*Lancet.*

